# Supplementary material for: Transcriptomic Analysis of Hormone Signal Transduction, Carbohydrate Metabolism, Heat Shock Proteins, and SCF Complexes before and after Fertilization of Korean Pine Ovules
Source: Int J Mol Sci. 2023 Mar 31;24(7):6570. doi: 10.3390/ijms24076570 (PMC10094794; doi:10.3390/ijms24076570)
Supplement: Supplementary file 1 [file ijms-24-06570-s001.zip › ijms-2308470-supplementary.pdf]

International Journal of
Molecular Sciences

MDPI

Supplementary Material

Supplementary Table S1. Statistics of reads quality after filtering

Sample	Total Raw Reads (M)	Total Clean Reads (M)	Total Clean Bases(Gb)	Clean Reads Q20(%)	Clean Reads Q30(%)	N(%)
ZA1	43.82	43.11	6.47	97.82	93.78	0.46
ZA2	43.82	42.95	6.44	97.88	93.97	0.49
ZA3	43.82	43.03	6.45	97.91	94.07	0.47
ZB1	43.82	42.79	6.42	97.95	94.16	0.92
ZB2	43.82	42.83	6.42	97.95	94.17	0.95
ZB3	43.82	42.72	6.41	97.9	94	0.94
ZC1	43.82	42.99	6.45	97.84	93.84	0.91
ZC2	43.82	42.92	6.44	97.85	93.9	0.93
ZC3	45.57	43.47	6.52	98.03	94.41	0.92
ZD1	43.82	42.77	6.42	97.82	93.83	0.93
ZD2	43.82	42.93	6.44	97.81	93.75	0.98
ZD3	43.82	42.91	6.44	97.9	94	0.96

Supplementary Table S2. Comparison results with Korean pine reference genome

Sample	Total Clean Reads (M)	Total Mapping(%)	Uniquely Mapping(%)
ZA1	43.11	92.77	62.86
ZA2	42.95	92.67	63.96
ZA3	43.03	92.33	62.35
ZB1	42.79	92.08	65.81
ZB2	42.83	92.16	65.72
ZB3	42.72	92.29	65.48
ZC1	42.99	92.06	65.14
ZC2	42.92	92.99	65.88
ZC3	43.47	91.69	65.66
ZD1	42.77	92.76	65.97
ZD2	42.93	92.19	65.09
ZD3	42.91	92.72	65.95

Supplementary Table S3. qRT-PCR primer sequences in Korean pine

Unigenes	Forward primer	Reverse primer
18s（Internal reference）	GCGGTGGTTTATGTGTTTG	GAAAGGGTTGAGGTAGGAAG
c154707.graph_c1 （Internal reference）	CAGCTCAGCTTGAAATGA	ACGAGTACTGTGAGGAAA
Pkor06G00725	CTCGTTGTCAAAGTGATCCT	AAATCCTTCCAAACCAACCT
Pkor09G02425	AAGAGGTTATGTGAGCTGACT	CCTTCAATCATCCTAGCAAGAG
Pkor05G01532	CGTCCTTAGCTGTGATGTT	CCATTGGAGGAGGTTGATT
Pkor10G02308	GCCAGATGAGGACATTACA	CCATTCTAGCAGTGTCATTG
Pkor01G00962	TCAAGAACTTGCTCGTATGT	GGATCATCTCCAACAAGAAGA
Pkor03G02356	CTCGGTTTGTGATAAAGACGGAAT	CATACTCCTTCTCTGCATCCTCTA
Pkor12G00743	CATCAGCAGCATCAGCAA	TTGTGGCATAAGCAGCAG
Pkor04G01664	GGAAATCACGAGGATACT	CTCTGCATCTCCATACAT
Pkor04G00304	AGGAACAGTTAGCAGACT	CCTCAAATTGAAGACCACT
Pkor01G02970	CTTAGAGGCAACACGAGAG	CTACACCAGCAGCAAGAA
